# Management of Distal Anterior Cerebral Artery Aneurysms in a Tertiary Center in Pakistan: A Retrospective Analysis of Treatment Modalities and Angiographic and Short-Term Functional Outcomes

**DOI:** 10.7759/cureus.104144

**Published:** 2026-02-23

**Authors:** Talha Sajid, Faiq Sheikh, Muhammad Naveed Majeed, Khawar Zaman, Muhammad Zahid Ullah, Ansarullah Khan, Muhammad A Zafar, Abdul Majid, Qasim Bashir

**Affiliations:** 1 Neurological Surgery, Punjab Institute of Neurosciences, Lahore, PAK; 2 Neurological and Neuroendovascular Surgery, Punjab Institute of Neurosciences, Lahore, PAK; 3 Clinical and Interventional Neurology, Punjab Institute of Neurosciences, Lahore, PAK

**Keywords:** anterior cerebral artery, intracranial, milrinone, subarachnoid hemorrhage, vasospasm

## Abstract

Background

Distal anterior cerebral artery (dACA) aneurysms are an uncommon subset of intracranial aneurysms and pose unique anatomical and technical challenges due to their deep midline location and complex vascular anatomy. While management outcomes are well described in high-resource settings, data from low- and middle-income countries (LMICs) remain limited, particularly in the context of treatment delays and resource-driven decision-making.

Objective

The objective of the study is to describe the treatment pathways, immediate angiographic occlusion rates, periprocedural complications, and short-term functional outcomes of dACA aneurysms managed with microsurgical clipping or endovascular coiling at a tertiary care center in Pakistan.

Methods

This single-center retrospective descriptive study included consecutive patients with radiologically confirmed saccular dACA aneurysms (A2-A5 segments) treated between January 2023 and July 2025. Patients underwent microsurgical clipping or endovascular coiling based on institutional practice pathways and clinical judgment. Angiographic outcomes were assessed using postoperative imaging, with Raymond-Roy classification applied for coiled aneurysms. Functional outcomes were evaluated using the modified Rankin Scale (mRS) at discharge and at the 30-day follow-up.

Results

A total of 49 patients were included, with a mean age of 47 years; 29 patients (59.1%) were female, and 47 patients (96%) presented with ruptured aneurysms. Microsurgical clipping was performed in 43 patients (87.8%), while six patients (12.2%) underwent endovascular coiling. The mean interval from diagnosis to definitive treatment was 18.2 ± 2.9 days, with no documented rebleeding prior to intervention. Complete aneurysm occlusion was achieved in 42 of 43 clipped aneurysms (97.7%) and in six of six coiled aneurysms (100%). At the 30-day follow-up, 44 patients (89.8%) were functionally independent (mRS 0-2), with no mortality reported.

Conclusion

In a resource-limited setting with significant treatment delays, definitive management of dACA aneurysms using microsurgical clipping or selective endovascular coiling achieved excellent immediate angiographic results and favorable short-term functional outcomes. These findings demonstrate the feasibility of managing this complex pathology within systemic constraints when treatment strategies are tailored to institutional capabilities.

## Introduction

Distal anterior cerebral artery (dACA) aneurysms represent an uncommon subset of intracranial aneurysms arising distal to the anterior communicating artery along the A2-A5 segments, accounting for approximately 1%-9% of all reported cases and presenting distinctive anatomical and clinical considerations due to their deep midline location within the interhemispheric fissure [1]. The pericallosal and callosomarginal junctions form the most frequent origin sites, where vessel angulation, branching patterns, and narrow operative corridors increase the technical complexity of both surgical and endovascular access [2]. These aneurysms are prone to rupture even at smaller sizes, often resulting in intraparenchymal hematomas and higher risks of neurological deterioration. Very distal variants involving the A4-A5 segments are exceedingly rare and frequently require tailored microsurgical strategies [3]. Although advances in catheter technology have broadened endovascular feasibility, treatment selection and outcomes remain influenced by aneurysm morphology and institutional expertise [4,5].

Existing outcome data for dACA aneurysms predominantly originate from high-resource centers where early presentation and hybrid capabilities are standard [6]. Long-term analyses from the International Subarachnoid Aneurysm Trial have highlighted the importance of treatment pathway selection and durable aneurysm occlusion in determining long-term survival and neurological recovery [7]. Furthermore, large clinical studies have demonstrated that early aneurysm treatment significantly improves functional outcomes and reduces disability compared with delayed intervention strategies [8]. Despite these advances, access to timely neurosurgical care remains uneven globally. Global neurosurgical workforce analyses have demonstrated that limited surgical capacity and healthcare infrastructure significantly restrict access to essential neurosurgical services in low- and middle-income countries (LMICs) [9]. Additionally, delays in securing ruptured aneurysms have been associated with increased pretreatment rebleeding risk and mortality, further emphasizing the importance of timely intervention [10].

To address this gap, this retrospective single-center study describes the management experience of distal ACA aneurysms within a tertiary neurosurgical center in Pakistan. The primary aim is to describe the angiographic occlusion results and periprocedural safety profiles associated with the two main treatment pathways employed: microsurgical clipping and endovascular coiling. A secondary aim is to report the short-term functional outcomes for the overall cohort. Documenting this experience may support context-sensitive clinical decision-making and resource planning in similar settings where management is shaped by both patient factors and systemic realities.

## Materials and methods

Study design and setting

This was a retrospective, descriptive study conducted at the Punjab Institute of Neurosciences, Lahore. All consecutive patients with radiologically confirmed saccular dACA aneurysms (A2-A5 segments) treated between January 2023 and July 2025 were reviewed to describe management pathways and outcomes.

Inclusion and exclusion criteria

Patients of any age with radiologically confirmed saccular distal ACA aneurysms involving the A2-A5 segments who underwent definitive treatment with microsurgical clipping or endovascular coiling during the study period were included, regardless of rupture status, provided complete clinical and imaging data were available. Patients with fusiform, dissecting, traumatic, infectious, or giant thrombosed aneurysms; cases lacking postoperative imaging; and patients previously treated for the same aneurysm were excluded from analysis.

Treatment modality selection and procedures

Treatment modality was selected based on existing institutional multidisciplinary practice pathways, which reflected resource availability and clinical judgment. Microsurgical clipping via an interhemispheric approach was the predominant and first-line treatment. Endovascular coiling was performed in patients considered higher risk for open surgery, as determined by factors including poor Hunt-Hess clinical grade at admission, advanced age, or explicit patient preference.

Vasospasm management protocol

All patients received standard oral nimodipine prophylaxis. During the preintervention waiting period, patients with ruptured aneurysms were managed according to a standardized departmental subarachnoid hemorrhage (SAH) protocol. This included strict euvolemic fluid management, controlled blood pressure optimization to reduce the risk of rebleeding while maintaining adequate cerebral perfusion, and close hemodynamic monitoring in a high-dependency or intensive care setting.

Clinical vasospasm was diagnosed based on new focal neurological deficits or deterioration in consciousness not attributable to hydrocephalus or rebleeding, supported by radiologic evidence of arterial narrowing on computed tomography angiography (CTA) or digital subtraction angiography (DSA) when available. Vasospasm was also strongly suspected in cases complicated by intraoperative aneurysm rupture.

A distinctive institutional protocol involved the use of milrinone. For patients undergoing endovascular coiling, milrinone was administered as an intra-arterial bolus during the procedure, given existing arterial access, followed by a continuous intravenous infusion for approximately 48 hours, based on evidence supporting its role in improving cerebral perfusion [11,12]. In the microsurgical cohort, intravenous milrinone was used therapeutically in patients who developed clinical signs of vasospasm in the postoperative period.

Occlusion assessment

Angiographic occlusion outcomes were assessed according to modality-specific standards. For endovascular coiling, aneurysm occlusion was evaluated using the Raymond-Roy occlusion classification [13]. For microsurgical clipping, occlusion was determined using postoperative imaging, including CTA or DSA, to confirm clip placement and vessel patency. Initial postoperative imaging served as the basis for outcome assessment in both treatment pathways.

Data collection and analysis

Relevant demographic, clinical, procedural, and outcome data were extracted from institutional medical records for the 49 included patients. Given the non-randomized treatment allocation and the small size of the endovascular cohort, this study was designed as a descriptive analysis. Continuous variables are presented as mean ± standard deviation, and categorical variables as frequencies and percentages. Data are presented separately for the microsurgical clipping and endovascular coiling pathways to illustrate the characteristics and outcomes associated with each as practiced in this setting. No inferential statistical comparisons between groups were performed.

Ethical considerations

This study was conducted in accordance with institutional ethical standards and maintained full patient confidentiality. Formal review was not required, as the study received an Institutional Review Board (IRB) exemption under No. 2322/IRB/PINS/Approval/2025, confirming that it involved retrospective analysis of existing clinical data without direct patient contact or intervention.

## Results

A total of 49 patients with distal ACA aneurysms were included in this descriptive analysis, treated between January 2023 and July 2025. The mean age of the cohort was 47 years, with a slight female predominance (59.1%). Microsurgical clipping was performed in 43 patients, whereas endovascular coiling was performed in six patients, reflecting institutional pathways and clinical selection criteria.

Most aneurysms were ruptured at presentation (47/49). Among these patients, the mean time from ictus to hospital presentation was 3.6 ± 2.4 days. The interval between radiological diagnosis and definitive intervention was 18.2 ± 2.9 days for the overall cohort, reflecting real-world constraints related to referral logistics and scheduling in a public-sector setting. No rebleeding events were documented during the waiting period prior to intervention. Clinical severity at admission was assessed using the Hunt-Hess grading scale. Most patients presented with good-grade SAH, with 35 patients classified as Hunt-Hess grade I-II, 10 patients as grade III, and four patients as grade IV. An associated intracerebral hemorrhage was identified in two patients at presentation.

**Figure 1:**
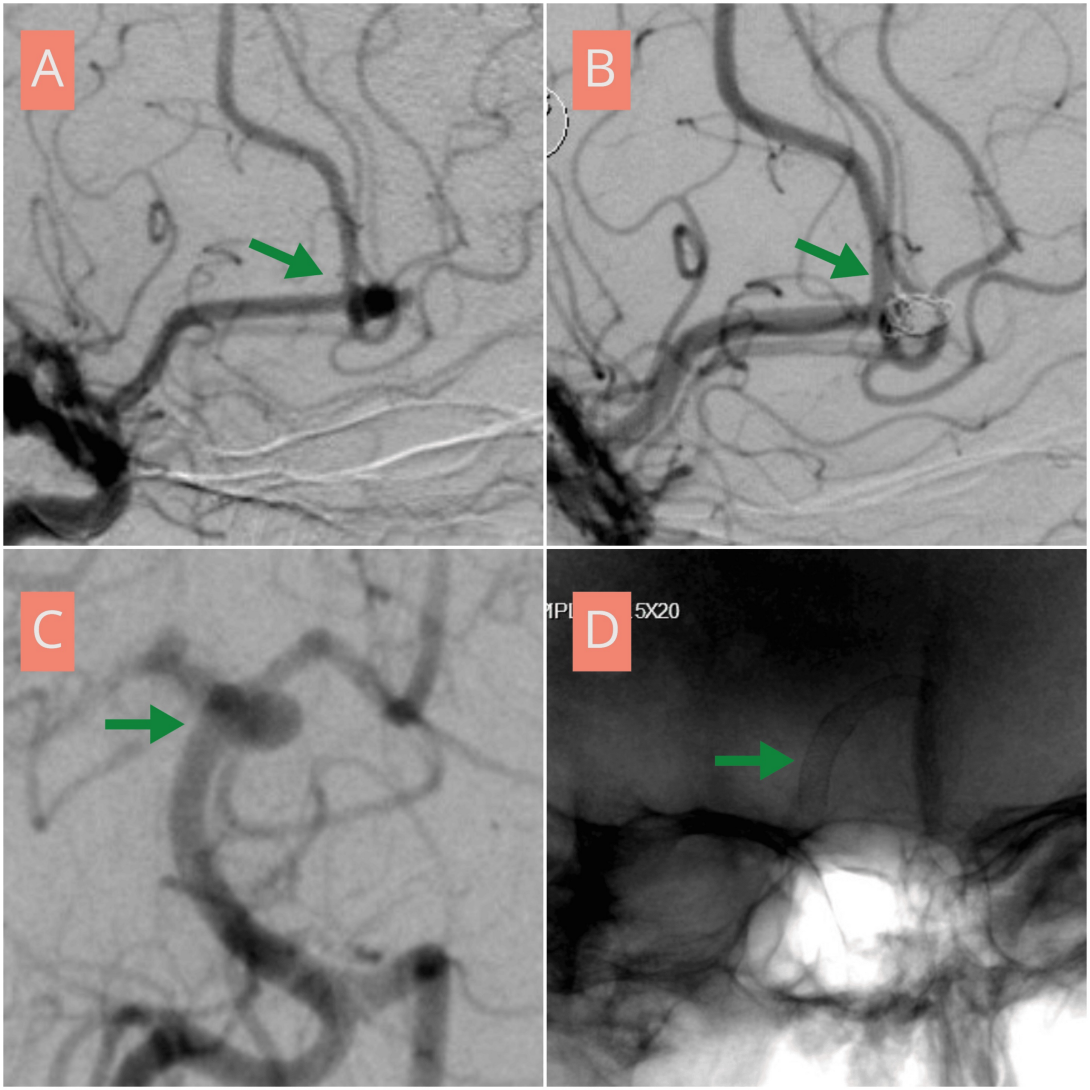
Angiographic demonstration of endovascular management of distal anterior cerebral artery and posterior circulation aneurysms in a 43-year-old female (A) Lateral view digital subtraction angiography (DSA) demonstrating a right anterior cerebral artery aneurysm at the A2-A3 junction (green arrow). (B) Post-embolization DSA showing complete obliteration of the aneurysm, consistent with Raymond-Roy class I occlusion (green arrow). (C) Oblique view DSA demonstrating a basilar artery aneurysm arising from the left posterior cerebral artery origin (green arrow). (D) Post-treatment angiogram showing deployment of a pipeline flow-diverter across the aneurysm neck (green arrow).

**Figure 2:**
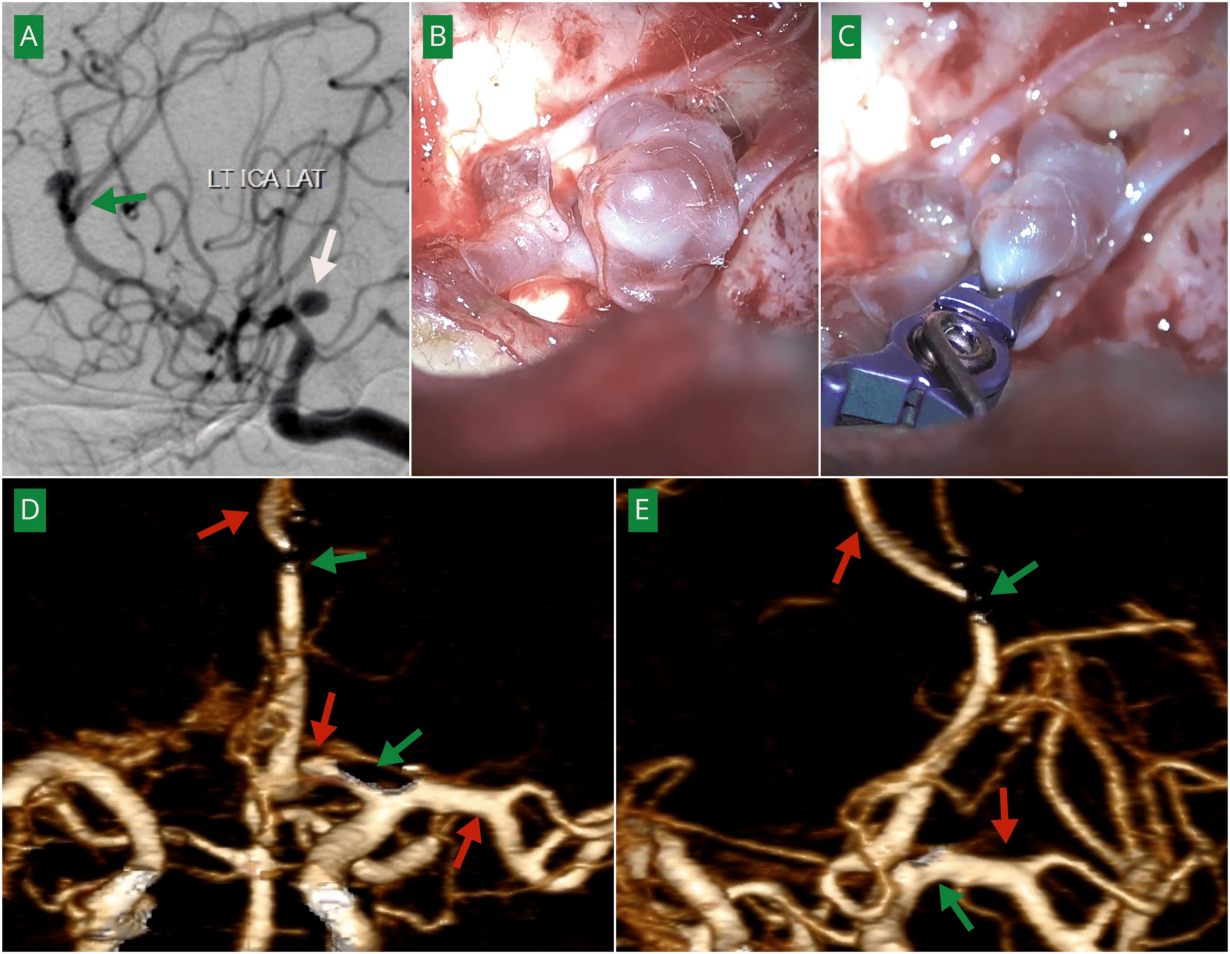
Angiographic and intraoperative images demonstrating microsurgical clipping of A2-A3 and ICA aneurysms in a 25-year-old male (A) Preoperative DSA showing a left distal anterior cerebral artery aneurysm at the A2-A3 junction (green arrow) and an internal carotid artery (ICA) bifurcation aneurysm (white arrow). (B) Intraoperative image demonstrating the aneurysm at the bifurcation of the A2 segment into A3 divisions. (C) Intraoperative image demonstrating clip placement with preservation of distal vessel patency. (D, E) Postoperative angiography demonstrating complete occlusion of both aneurysms (green arrows) with preservation of distal arterial flow (red arrows).

**Figure 3:**
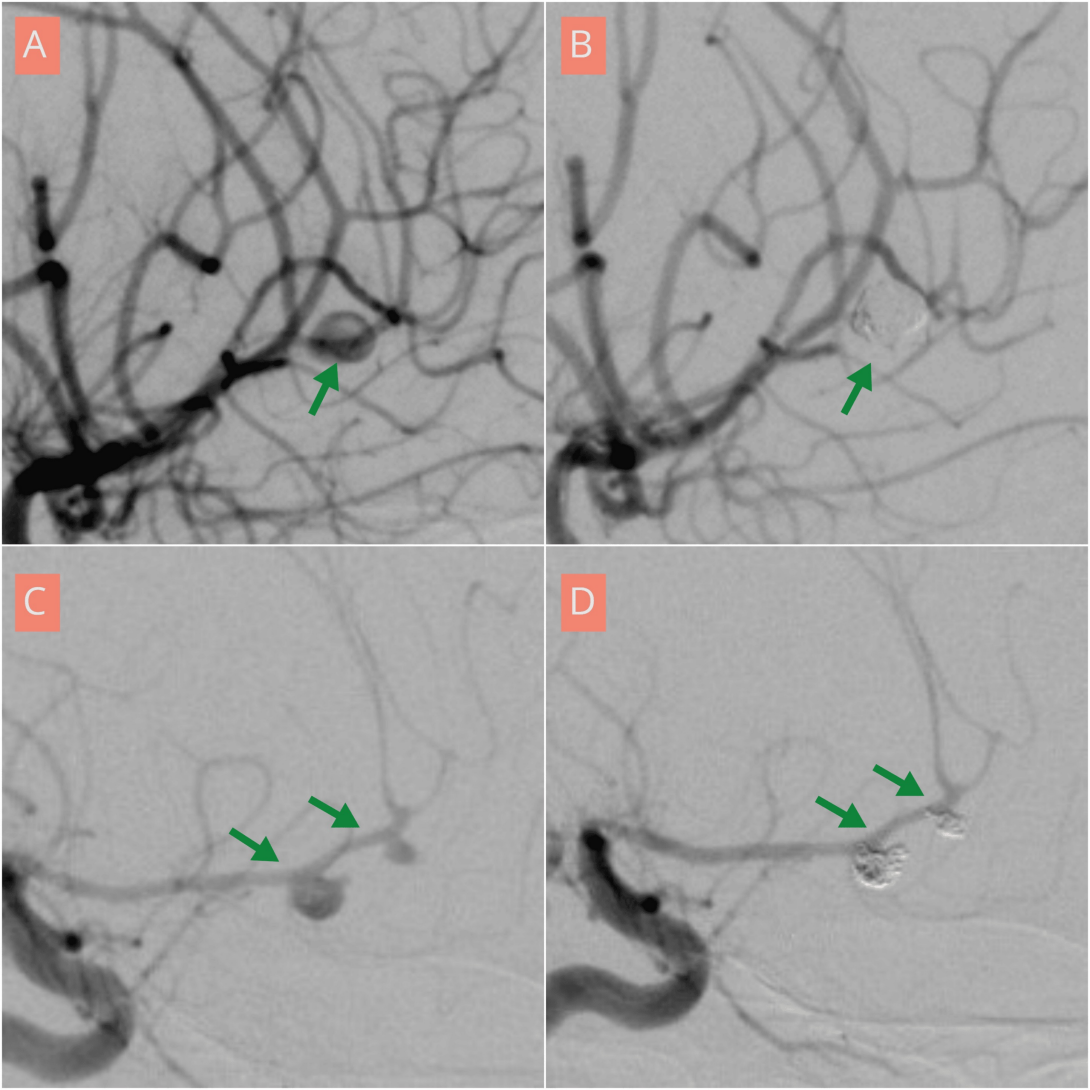
Angiographic demonstration of distal anterior cerebral artery aneurysms treated with endovascular coiling in a 30-year-old female (A and B) and in a 63-year-old female (C and D) (A, C) Lateral view digital subtraction angiography (DSA) demonstrating a right anterior cerebral artery aneurysm at the A2-A3 junction (A) and a left anterior cerebral artery aneurysm at the A2-A3 junction (C) (green arrows). (B, D) Post-coiling lateral view DSA demonstrating complete occlusion of the aneurysms (green arrows).

**Figure 4:**
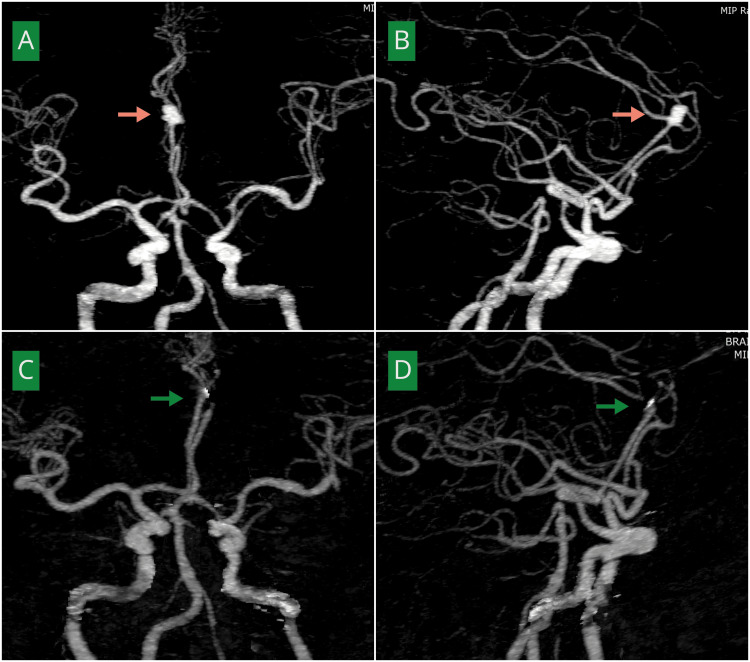
Preoperative and postoperative angiographic demonstration of microsurgical clipping of a distal anterior cerebral artery aneurysm in a 58-year-old male (A, B) Anteroposterior and lateral views of computed tomography angiography (CTA) of the brain demonstrating a right anterior cerebral artery aneurysm at the A2-A3 junction (pink arrows). (C, D) Post-clipping angiography of the same patient showing complete aneurysm occlusion with preserved distal filling of the anterior cerebral artery (green arrows).

Complex cerebrovascular anatomy was encountered in a subset of patients. In the coiling pathway, one patient underwent simultaneous coiling of a distal ACA aneurysm and a pipeline flow diverter for a basilar tip aneurysm (Figure 1). Another patient had multiple distal ACA aneurysms associated with a frontal arteriovenous malformation. In the clipping pathway, one patient harbored a distal ACA aneurysm and an ipsilateral internal carotid artery (ICA) bifurcation aneurysm, both clipped during the same craniotomy (Figure 2). Angiographic detail is given in Figures 3, 4.

Angiographic outcomes for each pathway were as follows: In the microsurgical clipping pathway, postoperative vascular imaging demonstrated complete aneurysm occlusion in 42 of 43 treated aneurysms (97.7%). In the coiling pathway, immediate post-procedural angiography confirmed Raymond-Roy class I (complete) occlusion in all six aneurysms. Occlusion status reflects the immediate postoperative imaging assessment.

Perioperative and postoperative complications were observed in the microsurgical cohort. These included two intraoperative aneurysm ruptures, postoperative hydrocephalus requiring ventriculo-peritoneal shunt (VPS) placement in three patients, seizures in two patients, new motor deficits in two patients, and wound infections in two patients. Motor deficits included both transient and persistent deficits. In the coiling pathway, one patient developed hydrocephalus, but no intraprocedural ruptures, new focal deficits, or wound issues were documented.

All patients received oral nimodipine. As per the institutional protocol, adjunctive milrinone therapy was administered in all six patients in the endovascular coiling pathway. In the clipping cohort, intravenous milrinone was administered to two patients who developed postoperative motor deficits attributable to vasospasm.

Functional outcomes, assessed by the treating neurosurgical team based on clinical documentation using the modified Rankin Scale (mRS), improved from discharge to 30-day follow-up across the cohort. For the overall series, 34 patients (69%) were independent (mRS 0-2) at discharge, improving to 44 patients (89.8%) by the 30-day follow-up; there were no deaths within 30 days. Within the microsurgical pathway, 41 patients (95.3%) achieved independence at 30 days. In the endovascular pathway, which was utilized for a select group of older, higher-risk patients, three patients (50%) were independent at 30 days.

Demographic, clinical, and outcome data for the microsurgical and endovascular treatment are described in Table 1.

**Table 1:** Demographic characteristics, clinical presentation, periprocedural variables, and short-term outcomes according to treatment modality *All patients in both groups received oral nimodipine as standard prophylaxis against vasospasm. Adjunctive milrinone therapy (intra-arterial bolus during the procedure followed by intravenous infusion for approximately 48 hours) was administered in all coiling cases and in two clipping patients who developed postoperative vasospasm-related motor deficits. SD: standard deviation; VPS: ventriculo-peritoneal shunt

Outcome/variable	Microsurgical clipping (n = 43)	Endovascular coiling (n = 6)
Mean age, years (mean ± SD)	45.6 ± 12.1	56.5 ± 8.0
Ruptured aneurysm at presentation, n (%)	41 (95.3%)	6 (100.0%)
Mean post-bleed day at presentation, days (mean ± SD)	3.6 ± 2.5	3.0 ± 1.8
Mean time from diagnosis to intervention, days (mean ± SD)	18.2 ± 3.0	18.2 ± 2.6
Intraoperative rupture, n (%)	2 (4.7%)	0 (0.0%)
Postoperative hydrocephalus, n (%)	3 (7.0%)	1 (16.7%)
CSF diversion required (VPS), n (%)	3 (7.0%)	0 (0.0%)
Postoperative seizures ("fits"), n (%)	2 (4.7%)	0 (0.0%)
New motor deficit (transient or permanent), n (%)	2 (4.7%)	0 (0.0%)
Wound infection, n (%)	2 (4.7%)	0 (0.0%)
Adjunctive milrinone therapy, n (%)*	2 (4.7%)	6 (100.0%)

## Discussion

Our single-center experience describes the management of dACA aneurysms within the specific context of a tertiary public hospital in Pakistan, highlighting the feasibility and constraints of a resource-limited neurosurgical environment. The principal observations from this descriptive analysis include a young patient cohort with predominantly ruptured presentations, significant pretreatment delays, high rates of immediate angiographic occlusion achieved via both employed modalities, a distinct complication profile associated with microsurgery, and encouraging short-term functional recovery facilitated by an aggressive vasospasm management strategy.

The demographic profile of our cohort-mean age of 47 years with a female predominance (60%) and 96% ruptured presentations-aligns closely with several key reports in the literature. Sharma et al. described a similar surgically treated cohort with a mean age of 54.8 years and 75% women, while Narang et al. also noted a female preponderance and a high rupture rate [14,15]. This consistency underscores that dACA aneurysms often manifest clinically with hemorrhage, a trend potentially amplified in settings like ours, where incidental detection is less common. In contrast, Take et al. reported an older population (mean 65.2 years), reflecting differing regional demographics [16].

A striking feature of our cohort is the interval from diagnosis to definitive treatment, averaging 18 days. Diagnostic angiography was typically completed within the first few days of admission; however, definitive intervention was delayed due to institutional capacity constraints, including operating room availability, limited endovascular suite scheduling, and the substantial patient burden faced by our center as the only specialized referral institution in the region. Additional limitations included restricted governmental resource allocation and intermittent shortages of essential surgical instruments, including aneurysm clips. This stands in contrast to protocols from high-resource centers advocating for ultra-early intervention. Early aneurysm treatment has been consistently associated with improved functional outcomes and reduced disability compared with delayed intervention [8]. Furthermore, analyses of the International Subarachnoid Aneurysm Trial have demonstrated that treatment pathway selection and timely aneurysm occlusion significantly influence survival and long-term neurological outcomes [7]. Evidence also suggests that delays in securing ruptured aneurysms increase the risk of pretreatment rebleeding and mortality [10]. The systemic factors underlying our observed delays reflect challenges inherent to public-sector neurosurgical services in many LMIC settings and are consistent with global reports highlighting restricted neurosurgical workforce capacity and healthcare infrastructure barriers [9]. Importantly, despite these constraints, we observed no periprocedural rebleeding and zero 30-day mortality, suggesting that diligent inpatient management of SAH during the waiting period may allow safe progression to definitive aneurysm treatment even in resource-limited environments.

The absence of documented rebleeding despite delayed intervention may be partially attributable to strict adherence to institutional SAH management protocols during hospitalization. Consistent nimodipine therapy, meticulous fluid balance, and blood pressure control likely mitigated early secondary complications while patients awaited definitive aneurysm treatment.

Consistent with institutional capabilities, microsurgical clipping was the dominant treatment pathway (43/49 patients). Endovascular coiling was selectively reserved for older patients or those with poorer Hunt-Hess grades-a selection pattern mirroring that described in other settings where coiling is chosen for anatomically suitable but clinically higher-risk cases [16]. Angiographically, both pathways excelled. The clipping pathway yielded complete occlusion in 97.7% of cases, aligning with more contemporary surgical series emphasizing meticulous microsurgical technique [15]. The coiling pathway achieved a 100% Raymond class I immediate occlusion rate, demonstrating technical feasibility in our setting, consistent with reports like those of Reference [4].

Economic considerations also influence treatment selection in LMIC settings. Advanced endovascular devices, including flow diverters, require individualized financial evaluation and were selectively utilized in carefully chosen cases within our cohort. Cost constraints remain an important contextual factor when interpreting management strategies in resource-limited environments.

The complication profile differed between pathways. Complications such as intraoperative rupture, new motor deficits, and wound infections were clustered in the surgical cohort, reinforcing the well-established procedural morbidity profile related to open surgery in this region. Conversely, the coiling pathway provided a less invasive alternative with fewer acute procedural complications, a finding supported by other analyses [6].

A distinctive aspect of our management strategy was the protocolized use of milrinone. In the endovascular pathway, milrinone was administered prophylactically as an intra-arterial bolus during the procedure, leveraging existing arterial access, followed by a short-term intravenous infusion in accordance with institutional protocol. In contrast, within the microsurgical pathway, intravenous milrinone was employed therapeutically in patients who developed clinical features suggestive of vasospasm. Although our study design does not permit conclusions regarding efficacy, the low incidence of permanent ischemic deficit (one patient) in a cohort experiencing substantial treatment delays is noteworthy. Our experience contributes to the growing body of observational evidence supporting further investigation of milrinone as a therapeutic adjunct in vasospasm management [11,12,17].

Despite delayed treatment, short-term functional outcomes were encouraging for the overall cohort, with 90% achieving independence (mRS 0-2) at 30 days and no mortality. The high rate of independence in the surgical group (95.3%) reflects its application as the primary treatment for most patients. The functional outcomes in the smaller coiling group, while less favorable, are interpreted in the context of this pathway being selectively offered to patients with higher baseline risk, a pattern of “confounding by indication” commonly described in similar real-world analyses [16]. Our results, along with global literature demonstrating the feasibility of achieving favorable outcomes despite systemic healthcare limitations, suggest that even with delayed access to care, favorable short-term recovery is possible through eventual secure aneurysm occlusion and proactive management of SAH complications [9,10].

Study limitations

This study inherits the limitations intrinsic to retrospective, single-center, descriptive designs. The small sample size, particularly for the endovascular cohort (n = 6), limits the generalizability of findings from this pathway. The most significant feature of the data is the profound treatment allocation bias, where clinical severity, age, and patient preference dictated modality choice, creating systematic baseline differences between the pathways; thus, outcomes are presented as descriptive of each pathway rather than comparative. Our follow-up duration is another constraint; the assessment at 30 days provides a short-term snapshot but offers no insight into long-term functional recovery, cognitive outcomes, or angiographic durability, particularly for coiled aneurysms, which carry a known risk of recurrence [6]. Furthermore, the study lacked systematic and objective monitoring of vasospasm, limiting our ability to rigorously evaluate the milrinone protocol. In addition, modified Fisher grading and aneurysm size were not consistently documented as predefined variables in retrospective records, precluding their inclusion in outcome analyses.

Future recommendations

Future work should focus on several key areas to build upon the findings described here. First, there is a pressing need for collaborative, multicenter registries in LMIC settings to pool data and better analyze how systemic factors like treatment delays impact long-term outcomes. Second, the role of adjunctive vasospasm therapies, including the protocolized use of milrinone as employed in our center, merits a dedicated prospective investigation to determine if such strategies improve outcomes compared to standard care. Finally, establishing structured, long-term follow-up protocols is essential to assess the durability of occlusion and the full long-term impact of treatment for dACA aneurysms.

## Conclusions

In conclusion, this descriptive analysis from a resource-constrained setting demonstrates that definitive management of dACA aneurysms is feasible despite systemic treatment delays. Microsurgical clipping served as the primary treatment modality, achieving high rates of occlusion and excellent short-term functional outcomes, albeit with a recognized profile of procedural complications. Endovascular coiling provided a less invasive treatment pathway for a selectively older and higher-risk patient subgroup. The overall encouraging short-term outcomes were supported by definitive aneurysm occlusion and an aggressive vasospasm management protocol that included the use of milrinone. These findings highlight the importance of adaptable treatment protocols that align with local expertise and resources. Larger, prospective studies with long-term follow-up are needed to further optimize management strategies for this complex pathology across diverse healthcare systems.
